# The London Hospital

**Published:** 1902-06-14

**Authors:** 


### THE LONDON HOSPITAL:

### QUARTERLY COURT.

A quarterly general and special court of governors was held at the London Hospital on Wednesday, June 4th, "to receive the report of the house committee for the past three months, and for other affairs."

The Hon. Sydney Holland was voted to the chair. Mr. G. Q. Roberts, the house-governor, was on his left, and some 20 governors, among whom was Sir Edmund Hay Currie, the newly appointed secretary of the Hospital Sunday Fund, sat round the room. The report did not take very long, and there were not many "other affairs."

### The Alterations.

As usual, the quarter just ended had been one of the most notable in the history of the London Hospital. The chairman said the governors would learn with satisfaction that the whole of the new floor, opened by the Lord Mayor so recently, was in full working order, and giving every satisfaction to the surgeons; with the exception of a few minor points, the whole of the centre block was finished. The main entrance opening into the new receiving-room, and the main corridor on the west side of the building, and the new staircase on the west wing, were also practically completed, and the children were occupying the wards on the first floor, which would be their own permanently, with the splendid balcony outside, where they would have the benefit of the fresh air, which was so necessary to their recovery. The re-arrangement of the east wing was proceeding, and it was the first time any wards had been covered with opalite tiles. Unfortunately the cost was so great (£960) that the committee felt it incumbent on them to refrain from extending the system, and the walls of the other wards would have to be covered with plaster. However, just before the meeting a gentleman had given £100 in order that the children's ward might have opalite tiles; they were so easily washed down with a sponge that, in a children's ward especially, their use was very valuable in the prevention of infection—bacilli could not so easily cling about glazed walls. Gloucester and Cambridge wards, where the tiles were, had been entirely refitted, new floors had been laid, and new ceilings made. Arrangements had been made for putting in two new boilers, with engineers' workshops, etc., and on the top of this wing a complete new hospital kitchen would be fitted up at a cost of £2,345.

An offer had been made to the Borough of Stepney to have the use of the new out-patients' hall for the King's Dinners; it would hold about 1,000 persons. It was hoped that this new department would be in use for its proper purpose before the next quarterly court was held. As soon as ever it was in use, the re-arrangement of the west wing would be proceeded with, new ophthalmic wards, small wards for the treatment of 24-hour cases, and a Coroner's Court, would be arranged for in the old out-patients' rooms. The wards above would be put into order and used again for the purpose for which they were being used at present, while on the top of all would be a complete set of Hebrew wards. The installation of the electric light was going on, and the Nurses' Home was being thoroughly painted and cleaned throughout at a cost of £930. Directions had been given with reference to a scheme for 260 nurses at a new home.

### A Nurses' Pension Scheme.

It had been agreed, Mr. Holland continued, that all the nurses should be eligible, after six years' service from the date of entering the training school, to an increase of £5 per annum on the present scale of pay. A second increase of £5 to be made after 12 years' service. At 45 years of age, and after 18 years' service a pension would be given, equal to the actual average pay of the previous five years; this pension would be continued during the pleasure of the committee, and special cases, not coming under the above qualifications, would be considered on their merits. The funds would be administered out of the endowments of the hospital. All those nurses who already belonged to the National Pension Fund would be given the opportunity of withdrawing from it if they desired to do so.

More Money Wanted.

The hospital, said Mr. Holland, was never in debt; it had invested funds to fall back upon, but during the last quarter the committee had not received the support it needed to deal with the heavy expenditure, and sales of stock had had to be effected to meet the demands. There was a balance of £28,000 on the wrong side. Notice had been received of a legacy, and he wished to press home the fact that more help of this kind was necessary. The Queen, in order to mark her appreciation of the work of the hospital, had signified her readiness to be made a patron.

Numbers and Efficiency.

Six hundred and sixty-four patients were in the wards, 728 being the highest number on any one day, and 625 the daily average for the quarter. That meant that the London Hospital was doing as much as any two hospitals in London.

Sir Edmund Hay Currie seconded the adoption of the report, and the meeting closed with the usual vote of thanks, proposed, after Mr. Holland's custom, by the chair.

